# An Essay on the Use of Nitric Acid, as an Escharotic, in Certain Forms of Hemorrhoidal Affections

**Published:** 1843-04-01

**Authors:** 


### BIBLIOGRAPHICAL NOTICES.

*An Essay on the Use of Nitric Acid, as an Escharotic, in Certain Forms of Hemorrhoidal Affections; illustrated by Cases.* By John Houston, M. D., M.R.I.A., Surgeon to the City of Dublin Hospital; Consulting Surgeon to St. Peter's Dispensary, and to the Deaf and Dumb Institution; Lecturer on Surgery at the School of Medicine, Park-street; Member of the Society of Naturalists and Physicians at Heidelberg, &c. &c.

[The Dublin Journal of Medical Science, &c. No. LXVII. Vol. XXIII. March, 1843.]

Caustic applications in the cure of hemorrhoidal affections, once in great favour with the profession, have of late years been generally abandoned, and their employment even denounced. A confidence, however, in one of the class, *nitric acid*, in some forms of this troublesome affection, induces Dr. Houston to recommend its use in the present publication. He does so, not only from his own experience, but also on that of many of his medical friends, among others Mr. Cusack, "who have found it a remedy at once efficient, safe, and easy of application."

In the common form of hemorrhoids, a simple varicose condition of the veins of the rectum, the surgeon is rarely applied to. It is only in their complications or consequences that his aid is generally sought. It is in the complication called "the vascular tumour" that Dr. H. considers the application of the nitric acid beneficial. He regards it as an affection of the mucous membrane and submucous tissue, and treats it as such. He believes that it usually has, as its basis, a knuckle or bunch of varicose veins; but that also it may be "a distinct and independent growth, the result of some other irritation in this region."

"I have seen it covering the surface of one varix in a rectum, while others in the same bowel have been smooth, and free from any such growth,—the former being the source of much annoyance, the latter giving no trouble at all. I have also seen the affection, in young individuals particularly, where the veins were quite free from any varicose dilatation, but in whom, after a time, varices formed as the result of the irritation of the vascular tumour. And I have observed that. in almost all cases of inward piles of long standing, no matter whether the affection have begun originally as a varix, or as a degeneration of the mucous membrane, both affections come to be present in conjunction, reciprocally aggravating each other's severity."

Their number varies from one or more; and in some cases they are so numerous as to cause, by "their protrusion through the anus, a permanently widened state of that aperture, and a habitual prolapse, not only of the tumour itself, but also of a portion of the bowel."

"In the early periods of the affection, the tumours are so soft, compressible, and free from pain as scarcely to be discoverable by the finger when introduced into the rectum, and scarcely, therefore, to be deserving of the term, tumour; but, when of long standing, and especially when they have been permitted to remain down for protracted periods at the water-closet, they acquire an increase of firmness and a tenderness which render them easily detected by such manipulations, and give them a tangible and permanent character. The dragging and pressure to which they are subjected in being pushed out and squeezed by the sphincter in defecation, renders them likewise prominent, and gives to them often a pedunculated or polypus-like form. The surface of the tumour is either granulated like a strawberry, or of a villous aspect. It is of a red colour, and, when protruded from the anus, bleeds from every pore as from a sponge. It is easy to satisfy one's-self on this latter head, by drying the surface of the tumour, when a fresh issue of blood instantly takes place from every point of its surface. The blood discharged, in such cases, is always of the arterial red colour,—a circumstance which often, in itself, indicates the true nature of the affection, and enables us to distinguish it from rupture of a varix. But, nevertheless, although this may be true as regards the direct issue of blood from the part, yet this very fluid may, if allowed to lie in the cavity of the rectum before being discharged per anum, acquire a dark red, and even a grumous character. The bleeding in the former is also of more frequent occurrence, appearing with every effort at defecation, and weakening the patient more by the frequency and persistency of the drain than by the quantity lost at stated periodical intervals, such as usually takes place in the bursting of varices from over-distension."

Dr. Houston believes that there are two varieties of organic lesions, which, though of different nature and origin, are equally productive of inconvenience, and susceptible of cure by the same means.

"The first is regarded by many as a sort of aneurism by anastomosis of the small vessels of the mucous membrane and submucous tissue exclusively, and may be independent from the first of varices of the general veins about the anus. Mr. Colles, who had an opportunity of examining the structure of one of those tumours in a person who died of another disease, says, "on slitting up the rectum, I saw three blood-vessels, each as large as a crow-quill, running for some way down the intestine and then dividing into a number of branches; these vessels ramified very profusely, and each seemed by interweaving of its branches to form one of these tumours. The trunks and branches were covered only by the lining membrane of the intestine. This examination shows us how inapplicable to this disease are the terms 'varicose tumors,' 'hemorrhoidal excrescences.'" This affection may occur in youth, and has been seen high up in the intestinal canal; but its most frequent seat is the lower part of the rectum. There is not originally or necessarily any pain arising out of it; but, by long exposure in the rectum to many sources of irritation, and by enlargement and prolapse from the anus, it runs into a state of actual disease. It differs from ordinary nævi in not being necessarily congenital, but resembles them too much in its persistent tendency to increase in growth. They are, both, affections which equally require operations for their removal.

"The second variety of the vascular tumour is of a chronic inflammatory nature, and may be best described by comparing it to the red, villous, tender, hemorrhagic surface exhibited by the mucous membrane of the eyelids in old cases of chronic conjunctivitis. With the latter, too, the resemblance is still farther established by its habit of secreting pus independently of ulceration. Such tumours are apt to form on old internal varices, which, by their projecting into the cavity of the bowel, expose the membrane covering them to more than ordinary pressure and irritation, and thereby become the direct cause of this morbid development. Once established in connexion with the surface of a varix, the two making between them a compound disease, the distressing qualities of such an affection are not slow in exhibiting themselves. Thus, in one case, in which there were several internal projecting varices, that only, on which this hypertrophied condition of the mucous membrane existed, gave annoyance, and on the removal of that and that singly, all hemorrhoidal distress subsided. The 'master pile' being removed, the others fell back into a state of painless quiescence. As in the foregoing variety, there is no relief for this affection but in destruction of the morbid growth. They both, therefore, differ from the diseased conditions which supervene on external varices, in having no disposition or power to undergo a spontaneous cure; and, consequently, are more likely, at some period of their course, to become objects of surgical interference."

The seat of the affection being then on the surface, is the use of the knife or ligature advisable? Is it not attended with risk, and is it not unnecessary? Should not such means be resorted to as will remove that surface without penetrating deeply, and risking the injury to large vessels! Our author thinks that the properties of *pure nitric acid* as an escharotic, point it out as singularly calculated to prove an efficient substitute for these severe remedies. The depth of the slough produced is easily regulated, and its lateral extension controlled by the immediate application of olive oil, which neutralizes its corrosive properties.

"The nitric acid, then, in its operation on the vascular tumour, combines in itself all the advantages possessed by excision or ligature, without any of their disadvantages. The tender, tumid, and bleeding surface is removed with little pain and without danger; and, in the cicatrization which rapidly follows, a radical cure is effected. But the good effects do not stop here; not only is a reparation of the worst part of the affection accomplished, but, by the bracing up of the general mucous membrane which follows the removal of the relaxed and diseased part of its surface, other varices which may be present are supported and reduced in bulk, and ulcers, or even fissures, are healed: and these secondary good results may be regarded as not the least important which have taken place on the occasion. The resumption of the natural action of the bowels, and the general improvement in health which follows the application of the acid to a single vascular hemorrhoid, even where several are left behind, show an improvement in the state of the rectum generally, greater than could be supposed to arise from the simple abstraction of one from among the number. The contraction of the mucous membrane following the removal of a portion of its extent, acts beneficially on the bowel, in the way, perhaps, that the contraction which follows the removal of it in the operation for prolapsus ani, acts; or in the way in which the removal of a piece of the scrotum, as recommended by Sir A. Cooper, operates in relieving varicocele; or, to use a still more familiar illustration, in that in which a laced stocking operates in remedying a varicose state of the veins of the lower extremities, viz., by bracing and giving support to the relaxed subjacent veins and other textures. The relief, too, which the bowel gains by the removal of such a source of local irritation, contributes, no doubt, in a prominent degree, to the production of the same good results.

"The application of the acid may be made in the following manner. Let the patient strain as at the night-chair, so as to bring the tumours fully into view; and, while they are so down, let him either lean over the back of a chair, or lie down in the bent posture on the side on which the disease exists, with the buttocks over the edge of the bed. Let a piece of wood, cut into the shape of a dressing case spatula, be dipped in the acid, and then, with as much of the acid adhering to it as it will carry without dripping, let it be rubbed on the tumour to the extent desired. The due effect of the acid on the part is shown by its changing it to a greyish-white colour. If a superficial slough be all that is required, a single application may be enough; if a more deep one, then two or three applications of the wood dipped in the acid may be made in quick succession; which being finished, let the part be well smeared over with olive oil, provided beforehand for the purpose. The prolapsed parts should then be pushed back within the sphincter, the patient put to bed, and an opiate administered. The pain of the application is sharp and burning at first, but goes off in two or three hours, and does not again return in the same form. A general uneasiness about the anus, on motion, together with a slight sense of heat, fulness, and throbbing, are felt for a few days; and there may be some little feverishness; but I have not seen or heard of any more serious effects from the remedy. In another case, a slight stranguary, which was experienced for a short time, disappeared under the mist. camphoræc. opio. The symptoms following the application of the acid are usually so mild as not absolutely to require confinement to bed more than a few hours; although, for many reasons, such confinement may often be desirable. On the third or fourth day, a purgative draught should be administered, when the bowels will be found to yield to the medicine, generally without either pain or prolapse of the rectum. The progress after this to healing is rapid, and free from any disagreeable symptoms."

A number of carefully detailed cases are appended, which, after an ineffectual resort to all the ordinary palliative modes of treatment, were successfully combatted by the new method. These cases are all such that, by the present established practice, the ligature would have been employed, and severe and protracted suffering would have inevitably resulted.

"One important point in recommendation of the treatment by nitric acid is, its immunity from danger and from pain, or subsequent tedious confinement; for, although in the cases above cited, severe pain and confinement for a few days to bed are spoken of with candour, yet these conditions are not to be taken as the measure of the results to follow in the generality of such cases. The anxiety felt for the success of a new remedy, by increasing my apprehensions regarding it, magnified the sufferings of the patients in my eyes, and impelled me to take precautions for their mitigation beyond, perhaps, their real urgency or necessity, and certainly beyond what I have since experienced in other cases, which, in the confidence of success, I have regarded with more indifference; for, in fact, I have since several times known it to happen, that, after the first feeling of pain, the effect of the caustic, had subsided, the patient has continued to transact his ordinary affairs as usual."

When two or more vascular tumours exist, Dr. Houston thinks that both should be touched at the same time. The circumstances of the case must here be our chief guide.

"But, let me not be misunderstood as to the cases for which I recommend the use of nitric acid. I do not speak of it as a remedy for all kinds and degrees of vascular tumours or internal hemorrhoids; nor as one to supersede entirely the knife or ligature. There are cases, no matter how they may have commenced originally, in which, from long standing or other causes, all the textures in the neighbourhood—blood-vessels, skin, mucous membrane, cellular tissue, &c., have become so implicated, that scarcely any one part appears worse than another; and in which some operation, if any, of a more sweeping nature must be had recourse to. For such, the knife or ligature may, according to the choice of the practitioner, be employed. The acid is, according to my experience, adapted especially for cases of more common, every-day occurrence; cases in which the disease, although not involving immediate danger, yet keeps individuals miserable, and interferes with them in the discharge of their ordinary duties of life; cases, in short, which astringents will not cure, and for which, excision or ligature would be unnecessarily severe remedies."

